# The Fellowship of Christmas

**Published:** 1920-12-25

**Authors:** 


### The Hospital

The Workers' Newspaper of Administrative Medicine and Institutional Life, Administration, National Insurance and Health.

No. 1803, Vol. LXIX. SATURDAY, DECEMBER 25, 1920. PRICE TWOPENCE

### THE FELLOWSHIP OF CHRISTMAS.

A preacher of our acquaintance was accustomed to begin his address on Christmas Day by saying that Christmas Day was not a day for sermons. This remark used to introduce, however, a very sensible discourse, in which one or other of the great lessons of the season was briefly emphasised. The really surprising thing, nevertheless, is the indifference of most preachers to the suggestions in which the Christmas festival is rich, and these we begin to discover when we ask what our loss would be if the festival ceased to be observed. In the first place, we should lose a traditional occasion for expressing one of the happy moods of mankind, and those who are indifferent to Christmas forget that these moods need more formal occasions for expression than their own uncertain recurrence provides. Rightly regarded, the Church's year, with its set feasts and fasts, represents the different phases of the drama of human life, and if these phases are to find symbolic expression they must be dramatised in their order, so that the experiences of each person can find expression in a common ritual, representing not only his own life but the life of mankind. The progress of the Church's year, then, provides the drama into the symbols of which each person can pair his own store of joy and sorrow. The succession of feasts and fasts, in which he is invited to share, provide a scheme into which his own moods can be ordered, so that these moods cease to be chaotic, but bind him to his fellows in a ritual common to himself and to his fellow-men. Were the feasts and the fasts suspended he would find his life imaginatively sterile, since the ups and downs of his own temperament would miss the channel into which both can be absorbed. The result of this suspension would be to reduce life to a grey monotony of days, indistinguished from one another, for a mood of joy or sorrow loses its imaginative satisfaction if it cannot be jointly shared. Our joys and sorrows can only become dignified if we are lifted beyond ourselves into the larger life of the race. This is the privilege which the Church's year confers, and we cannot be too grateful for it.

The fashion to treat Sundays like any other day of the week deprives the week itself of variety, and the same deprivation occurs when the feasts and fasts of the Church's year are neglected. For nature herself hates monotony as much as she hates a vacuum, and disdains a normal year in favour of the changing cycle of seasons, in which heat and cold, spring and autumn, abundance and death, succeed each other. This cycle of seasons lends to nature all her variety, and consequently all her charm. The perennial cold of the Polar regions, and the perennial heat of the tropics, are equally a kind of death; and there is much suggestion in the fact that the Sahara desert was produced by the too exuberant vegetation which preceded it. Since this cyclic change is the condition of all healthy life, it is natural that religion should reflect it and provide in the life of man a series of dramatic occasions in which all his moods are recognised in turn. The institutional side of religion is chiefly valuable for this reason. Not only does it provide satisfaction for his moods, but by giving ordered expression to them it reminds him of the permanent demands of his own nature. It follows from this that these demands receive their best satisfaction in institutions themselves, so that a school chapel, a hospital Christmas, even the nightly dinner in a college hall, are among the richest memories of all men. The common fellowship of institutional life reminds us all that we are members one of another, and that our life is rich in proportion to the ties by which it is bound to others. We should, therefore, expect that, just as the monastic orders have been famous for their hospitality and good cheer at the great festivals, so the Christmas spirit is seen most merrily in places like hospitals, much of whose work is busy with the sad scenes of pain, ill-health, and death. Were these not part of their business, Christmas would be less gay in them than it is; for, because of these scenes, the need for gaiety and cheerfulness is the greater; and fun and good cheer are the more genuine because of the dark background against which they are set. The Christmas festival also reminds those who work in hospitals that their prime concern is not disease but health, and that all study is wasted study which does not in the long run conduce to a healthier life for mankind.

It is commonly said that, as we grow older, Christmas is less happy than it used to be, and that it is really after all the children's festival. The answer to this objection is that the man and woman who have ceased to be children at heart are stunted creatures, and that, if we have no children of our own, we can at least go during Christmas to the places where children are found. It is unnatural to spend Christmas alone, and because it is unnatural the man who is alone at Christmas is unhappy. But since a lonely lot may fall to anyone, such a person should remember that the hospitals' doors are always open, and that he who has nowhere else to go will always be welcomed in a hospital. There everyone is busy and lively, and extra willing hands are always required, so that a stranger is wanted as well as welcomed in the great task of making others happy.

